# Synthesis, Characterization, and Biological Evaluation of Chitosan Nanoparticles Cross-Linked with Phytic Acid and Loaded with Colistin against Extensively Drug-Resistant Bacteria

**DOI:** 10.3390/pharmaceutics16091115

**Published:** 2024-08-24

**Authors:** Fabian Pacheco, Alejandro Barrera, Yhors Ciro, Dorian Polo-Cerón, Constain H. Salamanca, José Oñate-Garzón

**Affiliations:** 1 Grupo de Investigación en Química y Biotecnología (QUIBIO), Facultad de Ciencias Básicas, Universidad Santiago de Cali, Cali 760035, Colombia; fabian.pacheco01@usc.edu.co (F.P.); alejandro.barrera00@usc.edu.co (A.B.); yhors.ciro00@usc.edu.co (Y.C.); 2 Laboratorio de Investigación en Catálisis y Procesos (LICAP), Departamento de Química, Facultad de Ciencias Naturales y Exactas, Universidad del Valle, Cali 760001, Colombia; dorian.polo@correounivalle.edu.co; 3 Grupo de Investigación Biopolimer, Departamento de Farmacia, Facultad de Ciencias Farmacéuticas y Alimentarias, Universidad de Antioquia, Calle 67 No. 53-108, Medellín 050010, Colombia; 4 Grupo de Investigación Ciencia de Materiales Avanzados, Escuela de Química, Facultad de Ciencias, Universidad Nacional de Colombia Sede Medellín, Cra. 65 #59a-110, Medellín 050034, Colombia

**Keywords:** colistin, chitosan, phytic acid, bacterial resistance, nanoparticles

## Abstract

The natural evolution of microorganisms, as well as the inappropriate use of medicines, have accelerated the problem of drug resistance to many of the antibiotics employed today. Colistin, a lipopeptide antibiotic used as a last resort against multi-resistant strains, has also begun to present these challenges. Therefore, this study was focused on establishing whether colistin associated with chitosan nanoparticles could improve its antibiotic activity on an extremely resistant clinical isolate of *Pseudomonas aeruginosa*, which is a clinically relevant Gram-negative bacterium. For this aim, nanoparticulate systems based on phytic acid cross-linked chitosan and loaded with colistin were prepared by the ionic gelation method. The characterization included particle size, polydispersity index-PDI, and zeta potential measurements, as well as thermal (DSC) and spectrophotometric (FTIR) analysis. Encapsulation efficiency was assessed by the bicinchoninic acid (BCA) method, while the antimicrobial evaluation was made following the CLSI guidelines. The results showed that colistin-loaded nanoparticles were monodispersed (PDI = 0.196) with a particle size of around 266 nm and a positive zeta potential (+33.5 mV), and were able to associate with around 65.8% of colistin and decrease the minimum inhibitory concentration from 16 μg/mL to 4 μg/mL. These results suggest that the association of antibiotics with nanostructured systems could be an interesting alternative to recover the antimicrobial activity on resistant strains.

## 1. Introduction

Bacterial resistance is a serious global public health problem, leading to an alarming number of deaths annually. Approximately >700,000 people die annually because of infections caused by multidrug-resistant (MDR) bacteria, and this figure is projected to increase to 10 million deaths by 2050 [1]. Among the prominently resistant bacteria, *P. aeruginosa* stands out, categorized as a priority pathogen in the list published by the World Health Organization (WHO) [2]. *P. aeruginosa* is a Gram-negative bacterium commonly associated with nosocomial, immunocompromised host, and chronic infections in patients with structural lung diseases such as cystic fibrosis (CF) [3].

This microorganism is characterized by its several mechanisms of natural resistance to various conventional antibiotics and acquired resistance to antibiotics used in managing infections caused by MDR or extensively drug-resistant (XDR) bacteria [4]. This includes last-resort antibiotics such as colistin, a cationic lipopeptide that kills Gram-negative bacteria by binding to the negative charge of lipopolysaccharide (LPS) on the outer membrane of Gram-negative bacteria, modifying the permeability of the bacterial surface [5,6]. Additional potential mechanisms and targets of colistin action have been suggested, such as the induction of reactive oxygen species and the inhibition of respiratory enzymes [7]. Unfortunately, several resistance mechanisms against colistin have been recently identified. Colistin resistance has been reported to be mediated by the neutralization of the phosphate group (PO4-3) of the lipid A of LPS by adding 4-amino-4-deoxy-L-arabinose [8] and phosphoethanolamine [9] or increasing cardiolipin in the cellular membrane [10].

The increasing prevalence of colistin resistance has heightened the need to investigate novel formulations of this well-established antibiotic. Nanotechnology has emerged as powerful tool within the biomedical and pharmaceutical fields for combating antibiotic-resistant strains since the nanoparticles used as antibacterial agents can penetrate bacterial membranes and be good carriers of antibiotics [11]). For instance, the use of nanoemulsions conjugated with polymers has permitted the restoration of the biological activity of ampicillin against methicillin-resistant *Staphylococcus aureus* (MRSA) strains [12]. Another study reported a significant increase in the antimicrobial activity of peptides vehiculated within nanoemulsions coated with polymers [13]. Ciro et al. reported a decreased minimum inhibitory concentration (MIC) of ampicillin against β-lactamase-producing *S. aureus* and MRSA when vehiculated within nanoparticles composed of a biopolymer known as chitosan [14].

The drug–nanoparticle interaction is related to the association capacity with the drug and the physical stability of the system [15]. A strong drug–nanoparticle interaction provides adequate accessibility of the drug to the nanoparticle, meaning that the drug has a high loading capacity. In fact, drug–NP accessibility will further impact the distribution and migration of a drug in NPs [16]. For example, high drug accessibility to nanoparticles can be favorable for the physical stability of the formulation [17]. However, the magnitude of the interaction may be inversely proportional to the drug release capability, since high association efficiency will restrict drug diffusion. In this way, it is important to study the colistin–chitosan interaction. Differential scanning calorimetry (DSC) and infrared spectroscopy (FTIR), as sensitive instruments that provide reproducible data, have been used to determine the intensity of the interaction of antimicrobial peptides with other substrates [18,19] including polymers [20].

As a strategy to develop nanoparticles that can improve the performance of an antibiotic against resistant bacteria, the choice of a polymer with antimicrobial properties should be considered. Chitosan is a biopolymer obtained by the deacetylation of chitin; a polysaccharide extracted from crustacean shells. Because of its cationic charge, it exhibits antibacterial properties against sensitive *P. aeruginosa* [21] and XDR strains [22]. In the latter study, chitosan was incorporated into the surface of nanoemulsions and the antibiotic activity of colistin was not recovered against XDR bacteria. So far, the effect of chitosan nanoparticles against *P. aeruginosa* XDR is unknown.

Therefore, the research question arises: How does the antimicrobial activity of colistin against an extremely resistant clinical isolate of *P. aeruginosa* change due to its association with phytic acid cross-linked chitosan-based nanoparticles?

## 2. Materials and Methods

### 2.1. Bacterial Strains and Materials

Commercial chitosan (Mw = 477 kD) with a degree of deacetylation (DD) around 75%, phytic acid solution, sodium hydroxide, acetic acid, and colistin sulfate were acquired from Merck (Darmstadt, Germany). *P. aeruginosa* ATCC^®^ 27853™ was obtained from the American Type Culture Collection (ATCC; Rockville, MD, USA). *P. aeruginosa* XDR was isolated and phenotypically characterized according to Laverde et al. [22]. It was labeled as Pa03 MDR.

### 2.2. Chitosan Deacetylation

The deacetylation process followed the methodology outlined by Ciro et al. (2019), with some modifications [14]. A 10% (*w*/*v*) commercial chitosan suspension in 10 N NaOH solution underwent deacetylation in a microwave unit using 2 min warm-up cycles and a 20 min rest interval between each cycle. In total, 10 cycles were completed. Subsequently, the suspension was neutralized and vacuum-filtered, and the polymer was purified by washing with double-distilled water until a constant conductivity was achieved. Finally, the polymer was freeze-dried at −45 °C and 0.04 bar using a lyophilizer LF (Model FR-2016US, Rivadeneira Ingenieria SAS, Jamundi, Colombia).

The DD of chitosan was determined using infrared spectroscopy. Chitosan was mixed with KBr and compressed into a pellet. Then, the spectra were recorded on a Shimadzu FT-IR (IR Affinity-1S) between 4000 and 400 cm^−1^, with a 4 cm^−1^ resolution. The DD was calculated using the following Baxter equation:

(1) DD%=A1650A3450×115 

where 115 is the proportion of N-acetyl-glucosamine and N-glucosamine subunit molecular weights, and A1650 and A3450 represent type I amide and hydroxyl bands, respectively [23].

On the other hand, deacetylated chitosan was also characterized by proton nuclear magnetic resonance ^1^H-NMR, where the collected spectra were obtained by dissolving the polymeric material in D_2_O slightly acidified with acetic acid. Such spectra were recorded on a Bruker Ascend III HD spectrometer (Billerica, MA, USA) using a 5 mm TXI probe and operated at 600 MHz. MestRenova^®^ software (Mestrelab Research S.L., v.12) was used to collect the spectra. Likewise, this material was also characterized by Fourier transform infrared spectrophotometry-FTIR, as well as by differential scanning calorimetry-DSC. In the case of FTIR, 10 mg of the sample was used, which was mixed with KBr and where a JASCO ATR PRO450-S FTIR spectrometer (Hachioji, Tokyo, Japan) was used. While for the DSC, 5.0 mg of the sample was weighed into 90 µL aluminum crucibles TA Instruments, New Castle, USA) using an analytical balance (Sartorius^®^ MSE125P-100-DU, sensitivity 0.01 mg, Sartorius, Gottingen, Niedersachsen, Germany). The crucibles were hermetically sealed with aluminum lids and the analysis was performed using a Discovery SDT 650 DSC (TA Instruments, New Castle, DE, USA), in a temperature range from 25 °C to 300 °C at a heating rate of 2 °C/min. For the FTIR and DSC analyses, the chitosan precursor (Sigma Aldrich, San Luis, MO, USA) was used as a control and the respective spectra and thermograms are presented in the Supplementary File.

### 2.3. Preparation of Chitosan Nanoparticles Cross-Linked with Phytic Acid and Loaded with Colistin

**Figure 1:**
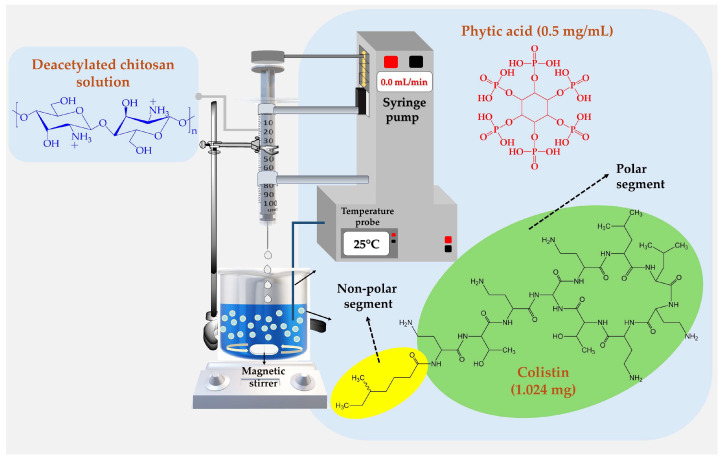
Schematic of the ionic gelation process for the preparation of nanoparticulate systems based on chitosan cross-linked with phytic acid and loaded with colistin.

A schematic representation of the development of chitosan nanoparticles cross-linked with phytic acid and loaded with colistin is illustrated in Figure 1.

For this NPs development, 0.5 mg/mL of phytic acid solution (in ultrapure water) and a 3 mg/mL chitosan solution (in 1% *v*/*v* acetic acid) were prepared independently. For the nanoparticle formation process, 3.0 mL of chitosan solution was slowly added from a 5 mL syringe with an injection rate of 0.5 mL/min into an aqueous solution containing the cross-linker phytic acid under magnetic stirring at 900 rpm at 25 °C, thus forming (Figure 1). The agitation was continued for an additional 5 min to ensure the formation of phytic acid–chitosan complexes through ionic gelation. Subsequently, 4.0 mL of these complexes were treated with an ultrasonic probe (CL-18, tip 4422) operated at 30% amplitude. Pulses of 30 s each followed by a 30 s resting time were employed for a total treatment duration of 4 min (blank-NPs). For colistin-loaded nanoparticles (CT-NPs), 1.024 mg of colistin sulfate was mixed with 5.0 mL of the phytic acid solution, and the same procedure was followed.

### 2.4. Physicochemical Characterization

#### 2.4.1. Determination of Colistin Aggregation by Tensiometric Analysis

Colistin aggregation was determined by means of surface tension studies, which was measured by the pendant drop method [24] using an OCA15EC^®^ drop tensiometer (Dataphysics Instrument, Filderstadt, Germany). For this, 500 µL of aqueous solutions of colistin with concentrations between 0 and 5 mg/mL were taken and deposited in a dosing syringe (SNP 165/119^®^, reference: 6000027), where the drop fall was recorded with a video camera coupled to the tensiometer. Thus, surface tension was determined by means of the Dataphysics SCA22 software, version 4.5.14. The measurements were carried out in triplicate at a temperature of 25 °C.

#### 2.4.2. Determination of Particle Size, Polydispersity Index-PDI, and Zeta Potential of Nanoparticulate Systems

The particle size, polydispersity index-PDI, and zeta potential of free colistin and chitosan, as well as phytic acid cross-linked chitosan NPs loaded and unloaded with colistin were analyzed using a Zetasizer nano ZSP (Malvern Instrument, Worcestershire, United Kingdom) equipped with a red He/Ne laser (633 nm). Dynamic light scattering (DLS) with a scattered angle of 173° at 20 °C and a quartz flow cell (ZEN0023) were used for measuring the particle size and PDI, while the zeta potential was measured using a capillary cell (DTS1070).

DLS measures the diffusion of particles under Brownian motion and uses a correlation function on the instrument corresponding to the Stokes–Einstein relationship. This permits researchers to obtain the particle size and size distribution parameters. The correlation function is obtained using cumulative data and is fitted to a simple exponential. From this, the mean size (z-average diameter) and an estimate of the distribution width (PDI) are acquired. Furthermore, the correlation function can fit a multiple exponential. From this, the particle size distribution is obtained as non-negative least squares or constrained regularization.

In this study, the particle size was described as the z-average diameter, and the PDI ranged from 0 to 1, which corresponded to a monodisperse and very wide distribution, respectively. Nanoparticle suspensions were prepared using 20 μL of the nanoparticle suspension and 980 μL of ultrapure water. Measurements were performed at 25 °C with an equilibration time of 2 min. Each sample was measured by triplicate, and the results were reported as means ± standard deviations.

#### 2.4.3. Characterization of Nanoparticulate Systems by DSC and FTIR

Free colistin and chitosan, as well as phytic acid cross-linked chitosan NPs loaded and unloaded with colistin were characterized by means of DSC and FTIR techniques, in a similar way as previously described in methodological Section 2.2. The amounts of colistin and free chitosan used were equivalent in proportion to those used in the preparation of nanoparticle systems. This was to establish whether there are changes in intensity or signal shifts that can be attributed to specific interactions between the components that make up the nanoparticulate system formed.

### 2.5. Encapsulation Efficiency

The determination of the encapsulation efficiency (EE) was carried out using 9.0 mL of aqueous dispersion of each nanoparticle system loaded with colistin, as well as unloaded (blank). Such NPs were centrifuged at 6000 rpm for 10 min, to subsequently take a 25 µL aliquot of the supernatant and deposit them in a 96-well plate system, where they were mixed with 200 µL of the bicinchoninic acid preparation (BCA method) [25]. Afterward, the plate was incubated at 35 °C for 30 min and the absorbances were read at λ = 562 nm, using a 800TS microplate reader (ThermoScientific Fisher, Waltham, MA, USA). The absorbance of the sample was compared against a previously obtained calibration curve in a range of colistin concentrations between 20 and 144 ppm (Y = 0.0008X + 0.2027, R^2^ = 0.9926). Thus, the encapsulation efficiency (EE) of colistin in chitosan NPs was calculated using the following equation:

(2) $$EE=\frac{Q_{t}-Q_{s}}{Q_{t}}\times 100$$

where *Q_t_* is the total amount of colistin used in the preparation of the NPs, while *Q_s_* is the amount of colistin obtained in the supernatant of the centrifuged dispersion.

### 2.6. Antimicrobial Activity

Bacterial sensitivity tests were performed following the guidelines described by the Clinical and Laboratory Standards Institute [26]. In brief, an overnight culture of *P. aeruginosa* ATCC 27853 and XDR strains (clinical isolate Pa03MDR, [22]) suspended in Mueller–Hinton broth was diluted with nutrient medium to achieve an optical density (at a wavelength of 625 nm) of 0.5 McFarland scale (~1 × 10^8^ CFU/mL). Subsequently, an additional dilution factor of 1/1000 was performed to reach a ~1 × 10^5^ CFU/mL concentration.

Fifty microliters (50 μL) of this bacterial suspension were mixed with 50 μL of free colistin and CT-Np independently at an adjusted initial concentration of 256 µg/mL. Then, serial dilutions were performed to achieve a final concentration of 0.5 µg/mL. Furthermore, serial dilutions with blank-Np were carried out, expressing the chitosan concentration. The serial dilution allows for obtaining the MIC known as the minimum concentration of the antimicrobial at which growth inhibition is observed.

### 2.7. Statistical Analysis

Minitab^®^ v.17 software (Minitab^®^ Inc., State College, PA, USA) was used to determine the impact on variables such as the particle size, PDI, and zeta potential resulting from the inclusion of colistin in phytic acid–chitosan nanoparticles. The Tukey post hoc test was used with a 95% confidence interval, and the data were presented as means ± standard deviations.

## 3. Results and Discussion

### 3.1. Chitosan Deacetylation

The chitosan deacetylation process occurred through alkaline treatment with sodium hydroxide, wherein the hydroxyl ion attacked the N-acetyl-glucosamine subunit and converted it to N-glucosamine. The assistance of microwaves facilitated a rapid and efficient process, achieving a deacetylation degree (DD) of 75% (commercial chitosan) to 95% (Supplementary File). Microwave heating directly interacts with the molecules in the reaction mixture, leading to a rapid temperature increase, reducing deacetylation time, and conserving energy [27,28]. Moreover, an increase in DD correlated with an enhancement in the antimicrobial activity of chitosan by contributing to a higher quantity of protonated amine groups interacting with the negative components of bacterial cellular membranes [28,29]. Furthermore, with a higher positive charge density on the chitosan backbone, smaller-sized nano systems can be obtained through ionic gelation [30].

### 3.2. Preparation of Chitosan Nanoparticles

Chitosan nanoparticles were obtained via ionic gelation, wherein electrostatic interactions between the protonated amine group of the chitosan backbone and ionized phosphate groups of phytic acid generated aggregates of phytic acid–chitosan. Ion–dipole interactions and hydrogen bonds further favored these aggregates [31]. Ionic gelation was chosen because of its simplicity, speed, and safety as a method for nanoparticle generation without using organic solvents, contributing to environmental conservation [32]. High-intensity ultrasound was used to decrease the size of the aggregates to the nanoscale, which led to large shear stresses and ultrasonic waves during sonication [33] A schematic of the possible way in which nanoparticles are formed is depicted in Figure 1.

### 3.3. Physicochemical Characterization

#### 3.3.1. Determination of Colistin Aggregation by Tensiometric Analysis

**Figure 2:**
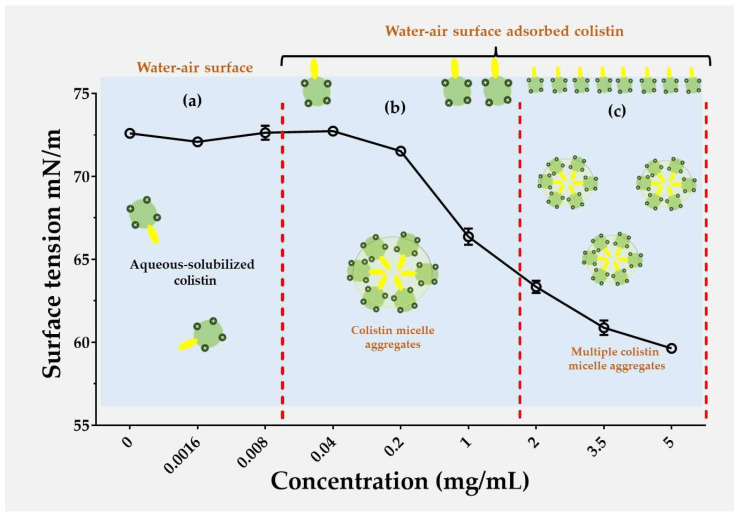
Characterization of surface tension of colistin in aqueous medium at different concentrations. (**a**) <0.008 mg/mL, (**b**) between 0.04 mg/mL and 5 mg/mL, (**c**) above 5 mg/mL.

The results of surface tension at different colistin concentrations are shown in Figure 2, where a typical profile of an amphiphilic molecule was found, which is consistent with the chemical structural of colistin.

In this way, colistin describes a dual polarity character in its structure, which consists of a polar segment based on multiple methylamino groups (which can be protonated in acidic medium), as well as a non-polar segment formed by an aliphatic alkyl substituent of seven carbon atoms (Figure 1). These results showed that at low colistin concentrations (<0.008 mg/mL), the surface tension tends to remain constant with a value around 73 mN/m at 25 °C (Figure 2a). This result suggests that at these concentrations, colistin is solubilized in the bulk solution with a minimum colistin amount in the surface zone. Nevertheless, the increase in its concentration from 0.04 mg/mL to 5 mg/mL leads to a reduction in surface tension from ~73 mN/cm to ~60 mN/m (Figure 2b). These results suggest that, at such concentrations, colistin begins to localize at the air–water surface, as well as in the bulk of the solution, where the colistin aggregation begin to take place, forming very small colistin–colistin aggregates, most likely of the micellar type. In contrast, above concentrations of 5 mg/mL, colistin multiple aggregates predominate (Figure 2c). These results are crucial to understand the way in which nanoparticulate systems are formed, as well as the encapsulation efficiency and antimicrobial effect achieved, as will be described in subsequent sections.

#### 3.3.2. Determination of Particle Size, Polydispersity Index-PDI, and Zeta Potential of Nanoparticulate Systems

The results of particle size, polydispersity index-PDI, and zeta potential of free colistin and chitosan, as well as the phytic acid cross-linked chitosan NPs loaded and unloaded with colistin are summarized in Table 1.

**Figure 3:**
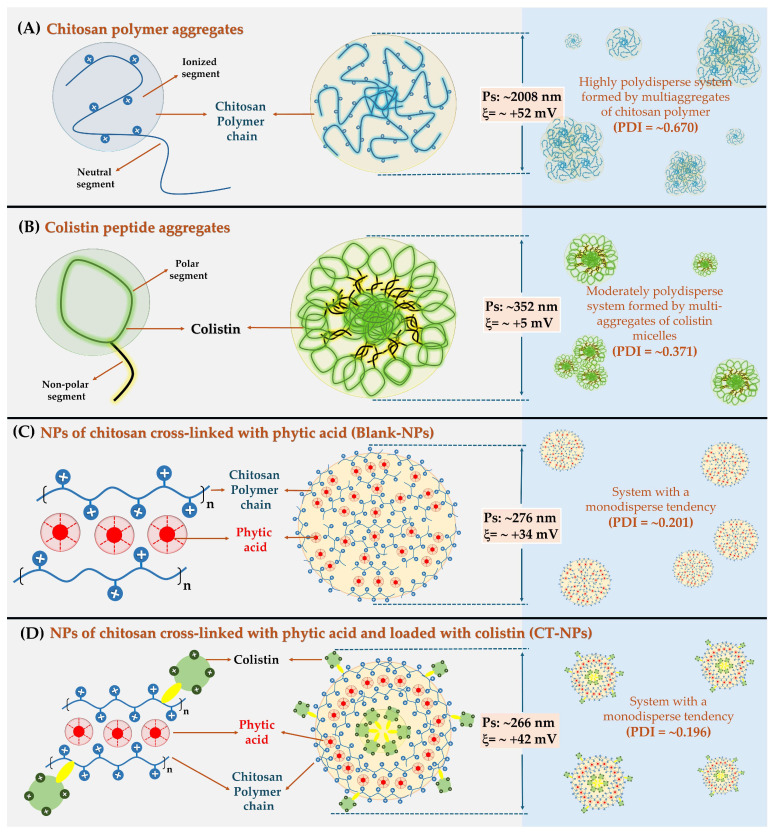
Schematic representation of the formation of aqueous dispersions of (**A**) chitosan aggregates, (**B**) colistin aggregates, (**C**) chitosan nanoparticles cross-linked with phytic acid (blank-NPs), and (**D**) chitosan nanoparticles cross-linked with phytic acid and loaded with colistin (CT-NP).

The results shown in Table 1 indicate that chitosan dispersed in an aqueous medium formed heterodisperse colloidal systems generated from the aggregation of multiple polymer chains and therefore, establishing high particle size (2008 ± 155 nm) and polydispersity (PDI = 0.670 ± 0.275) values. Such an aggregation phenomenon is mediated from the multiple inter-polymeric association of those polymeric neutral segments, while those ionized segments generate by the protonation of their amino groups provide it with a polycationic character (ξ: +52.4 ± 1.04 mV) (Figure 3A). In the case of colistin dispersed in water, a similar behavior was observed, where a polydisperse colloidal system with particle size of 351.8 ± 41.1 nm and a PDI of 0.371 ± 0.008 was achieved. These aggregation systems are probably formed by the multiple aggregation of micellar systems (Figure 3B). As previously described in Section 3.3.1, colistin can form micellar-type aggregation systems, which in turn can aggregate with each other due to its neutral surfactant characteristics evidenced by a low zeta potential value (+5.02 ± 0.65 mV). Nevertheless, these results are very interesting because they allow for demonstrating that the ionic gelation process entails the formation of nanostructured systems, which are mediated by the intrinsic characteristics of each material employed. In this way, the formation of blank-NPs is mediated by the cross-linking effect of phytic acid that forces the chitosan chains to organize in a highly structured manner (Figure 3C). In contrast, the formation of CT-NPs is mediated by the formation of micelles that lead to the cross-linked chitosan chains interacting with them, constructing a completely different type of particle (Figure 3D). All these results are very interesting since they demonstrate that the ionic gelation process leads to the formation of structured systems, which are mediated by the intrinsic characteristics of the compounds that make up the nanoparticle.

In the case of blank-Np, this exhibited a particle size of 276.5 ± 5.2 nm, whereas CT-Np displayed a particle size of 265.8 nm, indicating that the colistin load in the chitosan–phytic acid nanoparticles did not significantly affect their size. This is supported by a study conducted by Scutera et al., which reported similar sizes for blank and colistin-loaded albumin nanoparticles [34]. However, colistin may interact with phytic acid–chitosan complexes via electrostatic interactions, hydrogen bonds, and hydrophobic interactions such as London and van der Waals forces because of the primary amine, amide, carbonyl, and hydroxyl groups [35]. This interaction can increase the cross-linking between phytic acid–chitosan aggregates, resulting in a slight size reduction.

Muenraya et al. synthesized colistin–silver nanoparticles using a modified chemical method with sodium dodecyl sulfate as a stabilizer, resulting in sizes of 39.3–198.6 nm [36]. Dubashynskaya et al. developed succinyl chitosan–colistin conjugates with particle sizes of 114–180 nm and contributed to the amphiphilic character of the chitosan derivative and its self-assembled aqueous media [37]. Another study reported colistin-loaded alginate nanoparticles with a size of 111 nm, which was obtained using a top-down technique involving milling sodium alginate with zirconium oxide beads, reconstitution with Milli-Q water, and mixing with colistin solution in an ultrasonic bath [38].

The addition of colistin did not significantly affect the PDI of nanoparticles. The blank nanoparticles exhibited a PDI of 0.201 ± 0.020, whereas the colistin-loaded nanoparticles had a PDI of 0.196 ± 0.017 (Table 1). These values indicate homogeneous nanoparticle populations, likely facilitated by high-intensity ultrasound [31]. This agrees with Muenraya et al. who used sodium tripolyphosphate without ultrasound and obtained a PDI value of 0.975, indicating the formation of nonhomogeneous nanoparticle populations [37]. The homogeneous particle size is optimal for controlled colistin release and ensures greater physicochemical stability of nanoparticles.

Conversely, the zeta potential of blank and colistin-loaded nanoparticles was +33.5 ± 1.4 mV and +42.0 ± 1.7, respectively, indicating a positive net charge because of the higher chitosan concentration compared to the phytic acid concentration, which may be due to the possible location of some colistin molecules located on the surface of chitosan nanoparticles cross-linked with phytic acid. These results are consistent with Banoub et al. who obtained chitosan nanoparticles with a zeta potential of +37.7 mV, enhancing the antimicrobial susceptibility of antibiotic multidrug resistance and XDR isolates of *Actinobacter baumannii* [14]. Colistin slightly increased the zeta potential value, suggesting its entrapment in the nanoparticle surface. The primary amine groups of colistin can ionize in the slightly acidic media (5.0–6.0), wherein the nanoparticles were fabricated, enhancing interaction with the bacterial cellular membrane and increasing its antimicrobial effect [5].

#### 3.3.3. Characterization of Nanoparticulate Systems by DSC and FTIR

**Figure 4:**
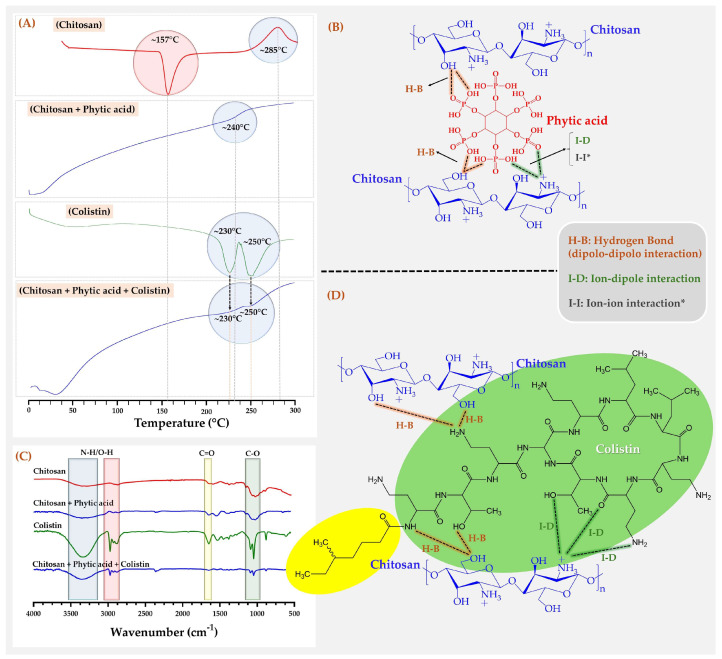
(**A**) DSC thermograms of NPs components, (**B**) schematic representation of colistin–phytic acid cross-linking chitosan, (**C**) FTIR spectra of NPs components, (**D**) schematic representation of colistin with a chitosan segment. * I-I interaction assumes that the phosphate group is ionized.

The results of DCS and FTIR characterization, as well as the schematic representation of the possible method of interaction between the components that make up the nanoparticulate systems are shown in Figure 4.

In the case of DSC analysis (Figure 4A), the usual signals of two thermal events for free chitosan corresponding to an endothermic melting peak at ~157 °C and a broad band at ~285 °C associated with the loss of amine groups in the main polymer chain [39] could be appreciated. It was also observed that polymeric cross-linking with phytic acid led to the disappearance of the exothermic melting peak, as well as the thermal signal of deamination. In contrast, the formation of a new signal at ~240 °C was observed, which was attributed to the formation of a possible glass transition of the new cross-linked material [40]. These results are consistent considering that the polymer chains of free chitosan have a higher degree of mobility when they absorb heat. On the contrary, when these polymer chains are cross-linked, they are more ordered (Figure 4B), increasing the capacity to absorb heat but with more restriction of movement. These results explain the loss of thermal signals of free chitosan at ~157 °C and ~285 °C, as well as the appearance of a new glass transition signal at ~240 °C. Hence, the increase in cohesiveness due to cross-linking leads to a greater heat requirement to melt the material, increasing its melting point. Furthermore, the cross-linking between the amino groups of chitosan and the phosphate groups of phytic acid makes them no longer available for deamination. Therefore, the formation of a new cross-linked complex structure triggers the formation of new configurations, which, in turn, generate glass transitions. These results can also be corroborated by means of FTIR spectra and specifically, with those related to the amino groups of chitosan (~3200–3300 cm^−1^) and phosphate of phytic acid (C=O at ~1750 cm^−1^ and O-H at ~2950 cm^−1^), which shift and change their intensity (Figure 4C), suggesting the formation of an association complex mediated by multiple thermodynamic equilibria between both compounds. Consequently, the amino group of chitosan acts as a weak base that entails the phosphate group ionization, establishing a system with multiple high-intensity ion–ion and ion–dipole interactions (Figure 4B).

Regarding colistin, it exhibited a similar behavior to those previously described, where two exothermic signals could be observed, one related to the fusion of pure colistin sulfate at ~230 °C and another at ~250 °C related to colistin degraded by the same heating DCS process [41]. Nevertheless, when colistin was combined with cross-linked chitosan, a considerable decrease in both signals could be observed, both by DSC (Figure 4A) and by FTIR (Figure 4C). These changes in DSC and FTIR signals can also be attributed to the formation of multiple high intensity interactions such as hydrogen bond, ion–dipole and ion–ion interactions (Figure 4D). Furthermore, it should be noted that in the case of phytic acid cross-linked loaded with colistin, a considerable decrease in the exothermic signals of colistin could be observed (~230 °C and 250 °C), as well as the loss of the glass transition signal of the cross-linked chitosan at ~240 °C, suggesting the formation of a new system where colistin is strongly associated with chitosan.

### 3.4. Encapsulation Efficiency

The colistin encapsulation efficiency in chitosan nanoparticles was 65.8 ± 8.9%. These values are found in the encapsulation efficiency reports of other studies with values of 67.0% [42], 76.4% [43] and 100% [44]. Colistin may be on the surface or inside the nanoparticulated system (Figure 3D). This is relevant to predict the release behavior of colistin that might be thought of in biphasic. First, there is a rapid release of colistin that is bound to the surface of the nanoparticle through electrostatic interactions and hydrogen bonds, favored by the concentration gradient [31,43]. Subsequently, controlled release of the colistin found within the nanoparticle is given by erosion and swelling phenomena of the chitosan–phytic acid complexes [31].

### 3.5. Antimicrobial Activity

Bacterial sensitivity tests were performed to determine if colistin associated with chitosan nanoparticles exhibits enhanced antibacterial performance against sensitive and XDR *P. aeruginosa* bacteria.

**Figure 5:**
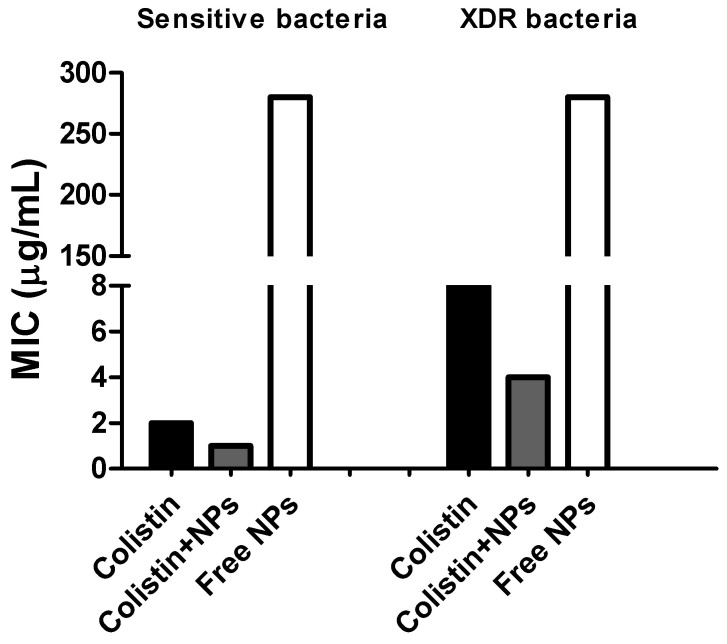
Minimum inhibitory concentration by microbroth dilution method of free colistin (black color), colistin associated with NPs (grey color), and colistin-free NPs (with color, concentration of chitosan) in sensitive and XDR *P. aeruginosa* Bacteria.

In sensitive bacteria, colistin revealed a MIC of 2 µg/mL (Figure 5). This was consistent with the established inhibitory concentration range for sensitive *P. aeruginosa* [45] Colistin, a cationic lipopeptide, initially interacts with the lipid A of the lipopolysaccharide of Gram-negative bacteria. Subsequently, colistin is introduced to the membranes via self-promoted absorption, causing irreversible alterations in phospholipid organization and ultimately killing the bacteria [6]. In the case of colistin associated with chitosan nanoparticles, the MIC was reduced by half, suggesting an additional antimicrobial contribution from NP. The NPs are used as antimicrobial agents due to their ability to penetrate bacterial membranes, disrupt biofilm formation, and be good carriers of antibiotics [11]. On the other hand, the contribution of chitosan to this result cannot be neglected, since this polymer has been widely known to act on anionic microbial membranes via electrostatic interaction, the additional antimicrobial contribution to the colistin effect could result from chitosan-mediated inhibition of bacterial transcription [46] Furthermore, chitosan’s flocculation of electronegative elements interferes with bacterial physiological activities, leading to bacterial cell death [47]. Moreover, chitosan’s chelating ability toward essential metals for bacterial survival affects cell viability [48].

The NP also exhibited antimicrobial activity against sensitive strains at a chitosan concentration of 280 µg/mL (Figure 5), confirming that chitosan without colistin possesses antimicrobial properties. This suggests that the electrostatic force between chitosan and the bacterial cell wall promotes a closer interaction with charged molecules, leading to nanoparticle infiltration through the bacterial cell wall. This increases the likelihood of nanoparticle accumulation at the interaction site and subsequently destabilizes the membrane [49]. Despite the pore size of the *P. aeruginosa* cell wall being 13 ± 5 nm [35], and the nanoparticle size being 276.5 nm, it could be assumed that it disassembles on the bacterial surface, releasing extended chitosan particles, as previously discussed. However, some possibilities could explain this phenomenon: (i) the cell wall is a dynamic structure that may vary during cell replication, permitting the entry of larger components [50]; (ii) the cell walls of some microorganisms are capable of remodeling, and their viscoelastic properties contribute to the migration of larger molecules through their surfaces [51]; (iii) certain compounds, such as dithiothreitol (DTT) and Ethylenediamine tetra acetic acid (EDTA), can increase the pore size of the cell wall [52], as well as polymers like polyethylene glycol [50]. Thus, chitosan may possess this ability.

With respect to the resistant bacteria, the MIC exhibited by free colistin was 8 µg/mL (Figure 5). This result was consistent with Humphries et al. [30], who reported that the *P. aeruginosa* strain, resistant to colistin, had an MIC of >4 µg/mL. Laverde et al. [22] reported that this strain is resistant to all conventional antibiotics, although the molecular mechanism of resistance is unknown. Generally, such resistance is attributed to the decrease in the negative surface charge density of the bacterial surface because of structural modifications of lipopolysaccharide lipid A or anionic phospholipids such as phosphatidylglycerol [53]. A modification of the lipid composition of the membrane in *P. aeruginosa* bacteria resistant to colistin has also been reported [10]. However, only recently it was understood, how the alteration of the lipid composition could be associated with resistance [5]. Thus, modification of lipid A or alteration of membrane phospholipids are responsible for requiring a higher amount of colistin to kill a resistant bacterium. Subsequently, when colistin was associated with the chitosan NP, the MIC was halved, suggesting that with high probability, chitosan would be acting on intracellular targets by interfering with essential physiological processes for cell survival, as discussed previously.

In contrast to what was reported by Laverde et al. [22], wherein chitosan did not have an additional antimicrobial contribution to the effect exhibited by colistin, we observed a slight antimicrobial contribution in our study. This can be explained from two perspectives: (i) the amount of chitosan that was associated with the surface of liposomes elaborated by Laverde et al. [22] was less than that used in these NPs (in this study); (ii) the spatial organization of chitosan being ionically associated with a negative element favored its orientation toward bacterial action targets. This is attributed to the fact that there is a high probability that chitosan does not act directly on the surface of colistin-resistant bacteria since they have a lower negative charge density. Rather, they would have the ability to translocate the surface, internalizing within the cell, affecting RNA synthesis, flocculating organelles or chelating essential metals for cellular physiological processes [49,54].

## 4. Conclusions

Chitosan was deacetylated in >95% with microwave assistance. Chitosan NPs loaded with colistin were prepared by ionic gelation assisted with high-intensity ultrasound. The size of the obtained NP was within a nanometric scale, and the NP population had a homogeneous size. Neither of the variables underwent significant changes before and after colistin encapsulation. The Z potential of the nanoparticle-loading colistin was significantly higher than that of the free NP. The results obtained by DSC and FT-IR show a pronounced non-covalent interaction between colistin and chitosan nanoparticles consistent with an encapsulation efficiency above 50%.

Regarding antimicrobial activity, the MIC of colistin was halved after being encapsulated within the NPs, in sensitive and XDR bacteria, suggesting a relevant antimicrobial contribution of chitosan NPs to colistin. Furthermore, the free colistin NP exhibited antimicrobial activity against *P. aeruginosa*, regardless of whether it was or was not resistant to colistin.

Emerging research addressing the recovery of the biological activity of antibiotics in multidrug-resistant bacteria positively impacts public health. This is because it strengthens the design and development of new strategies to overcome the bottleneck that exists between the time required for the discovery of new antibiotics and the rapid rate of bacterial resistance dissemination worldwide.

## Figures and Tables

**Table 1:** Physicochemical characterization of free colistin and chitosan, as well as phytic acid cross-linked chitosan NPs loaded and unloaded with colistin.

System Dispersed in Water	Physicochemical Parameter		
	Particle Size (nm)	PDI	ξ (mV)
Free chitosan	2008 ± 155	0.670 ± 0.275	+52.4 ± 1.04
Free colistin	351.8 ± 41.1	0.371 ± 0.008	+5.02 ± 0.65
chitosan NPs cross-linked with phytic acid (blank NPs)	276.5 ± 5.2	0.201 ± 0.020	+33.5 ± 1.4
chitosan NPs cross-linked with phytic acid and loaded with colistin (CT-NPs)	265.8 ± 4.5	0.196 ± 0.017	+42.0 ± 1.7 ^a^

^a^ indicate a statistically significant difference (*p* < 0.05). PDI: Polydispersity Index, ξ: zeta potential.

## Data Availability

The original contributions presented in the study are included in the article/Supplementary Material, further inquiries can be directed to the corresponding authors.

## Supplementary Materials

The following supporting information can be downloaded at: https://www.mdpi.com/article/10.3390/pharmaceutics16091115/s1, Figure S1: 1HRMN spectra of chitosan with high degree of deacetylation (DCH); Figure S2: FTIR spectra for the commercial chitosan (Aldrich) and processed chitosan with high degree of deacetylation (DCH); Figure S3: DCS Thermogram for commercial chitosan (Aldrich) and processed chitosan with high degree of deacetylation (DCH); Figure S4: Calibration curve of colistin for calculating the encapsulation efficiency.
