# Supplementary material for: Synthesis, Characterization, and Biological Evaluation of Chitosan Nanoparticles Cross-Linked with Phytic Acid and Loaded with Colistin against Extensively Drug-Resistant Bacteria
Source: Pharmaceutics. 2024 Aug 24;16(9):1115. doi: 10.3390/pharmaceutics16091115 (PMC11435368; doi:10.3390/pharmaceutics16091115)
Supplement: Supplementary file 1 [file pharmaceutics-16-01115-s001.zip › pharmaceutics-3034889-supplementary.pdf]

Evaluation of the antimicrobial activity of Colistin loaded in chitosan-phytic acid nanoparticles against extremely drug-resistant bacteria

Fabian Pacheco [1], Alejandro Barrera[1], Yhors Ciro[1], Dorian Polo[2], Constain H. Salamanca[3,4,*]and José Oñate-Garzón [1*]

Supplementary material

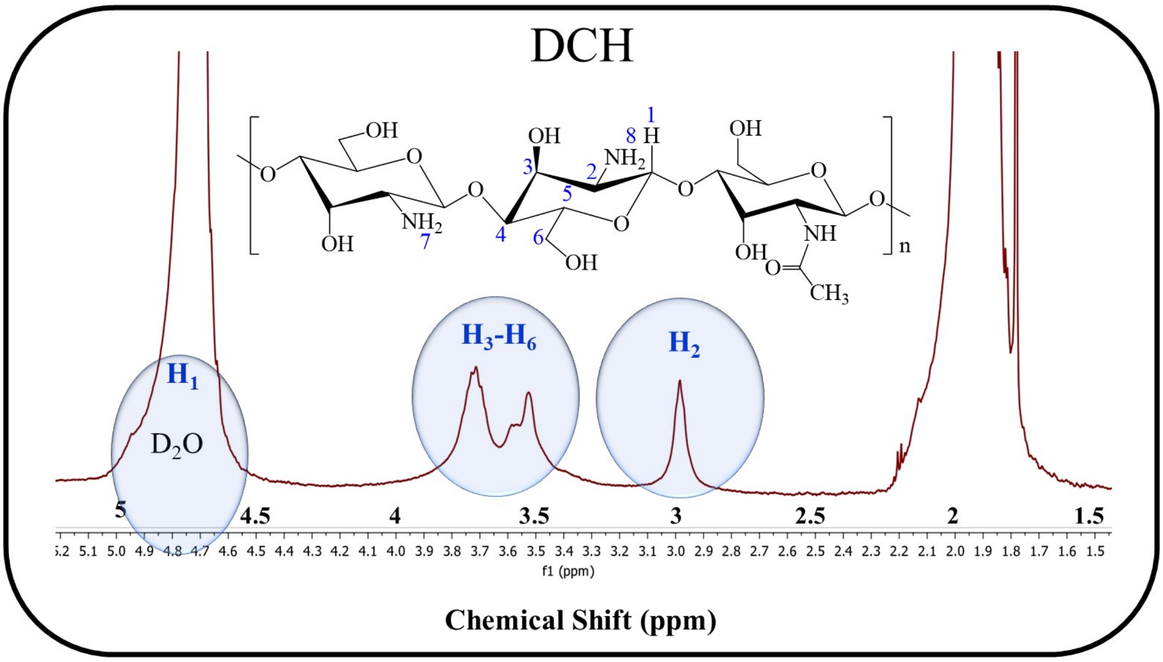

Figure S1: ^{1}HRMN spectra of chitosan with high degree of deacetylation (DCH)

The DCH spectrum shows signals at 2.9-3.1 ppm attributed to the hydrogen proton of the glucosamine subunit. The peaks at 3.4-4.0 ppm represent the H3-6 anomeric protons of the carbohydrate ring. The signal corresponding to H1 (not shown in the spectrum) is obtained at a chemical shift between 4.8-5.0, overlapping with the D_2O signal.

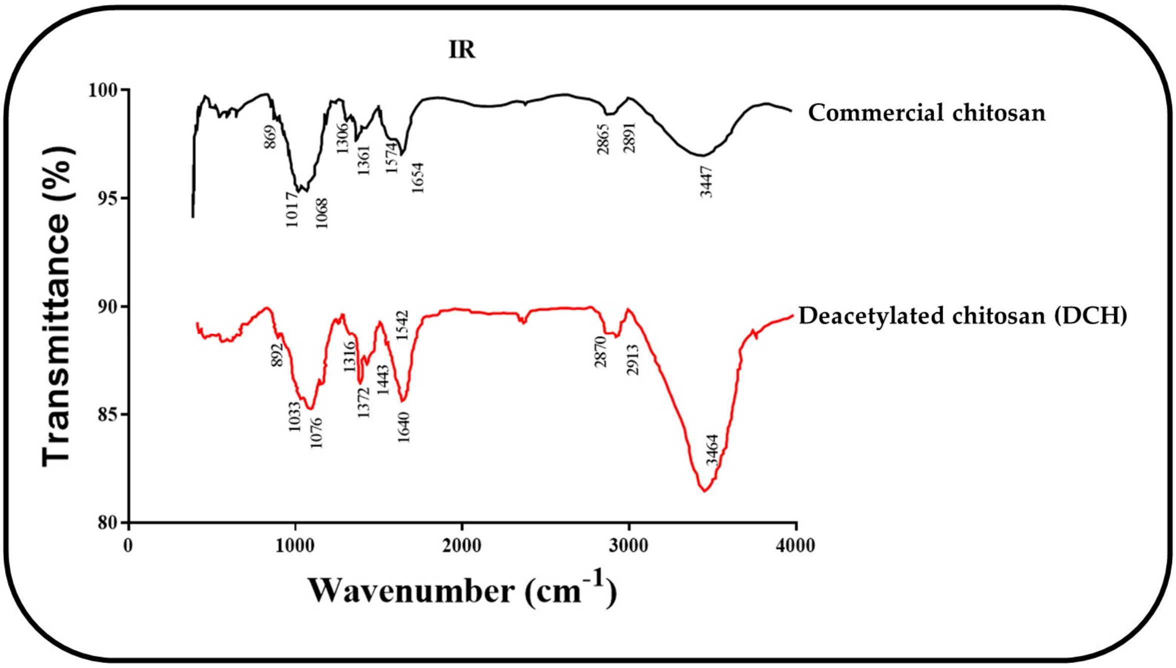

Figure S2: FTIR spectra for the commercial chitosan (Aldrich) and processed chitosan with high degree of deacetylation (DCH)

FTIR spectra of DCH showed characteristic peaks at 3464 cm^{-1}, 2913 cm^{-1} and 2870 cm^{-1} which are attributed to OH vibrations and C-H stretching, respectively. Moreover, it showed vibration bands at 1654 cm^{-1} due to C=O stretching, and a shoulder due to the deacetylation process at 1542 cm^{-1} attributed to the deformation of primary amine. Likewise, the peaks at 1443 cm^{-1},1372 cm^{-1} and 1316 cm^{-1} are ascribed to CH_2 bending and CH deformation, C-CH_3 stretching, and secondary amide stretching, respectively. Further, the vibration signals at 1076 cm^{-1}, 1033 cm^{-1} and 892 cm^{-1} are attributed to the ring C-O-C stretching, CO stretching and the C-O-C linkage stretching, respectively.

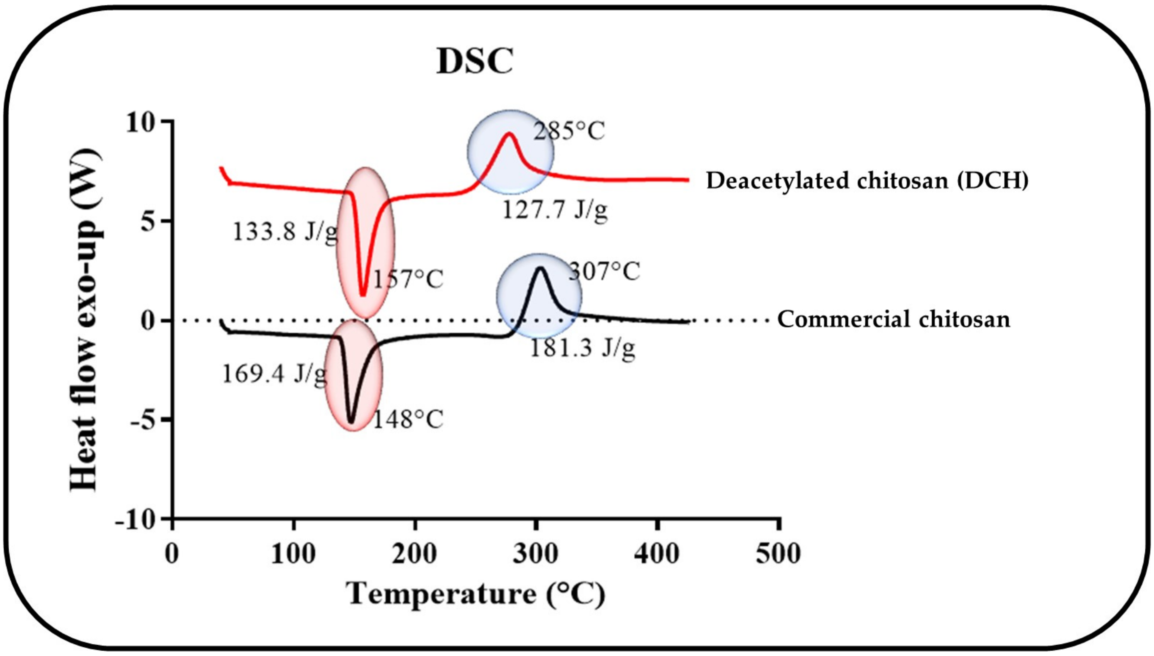

Figure S3: DCS Thermogram for commercial chitosan (Aldrich) and processed chitosan with high degree of deacetylation (DCH)

The thermogram of DCH polymer presented an endothermic peak at 157°C, which was attributed to the melting temperature. In addition, the exothermic peak was shifted to 307°C due to the loss of amine groups of the chitosan backbone and partial depolymerization of chitosan.

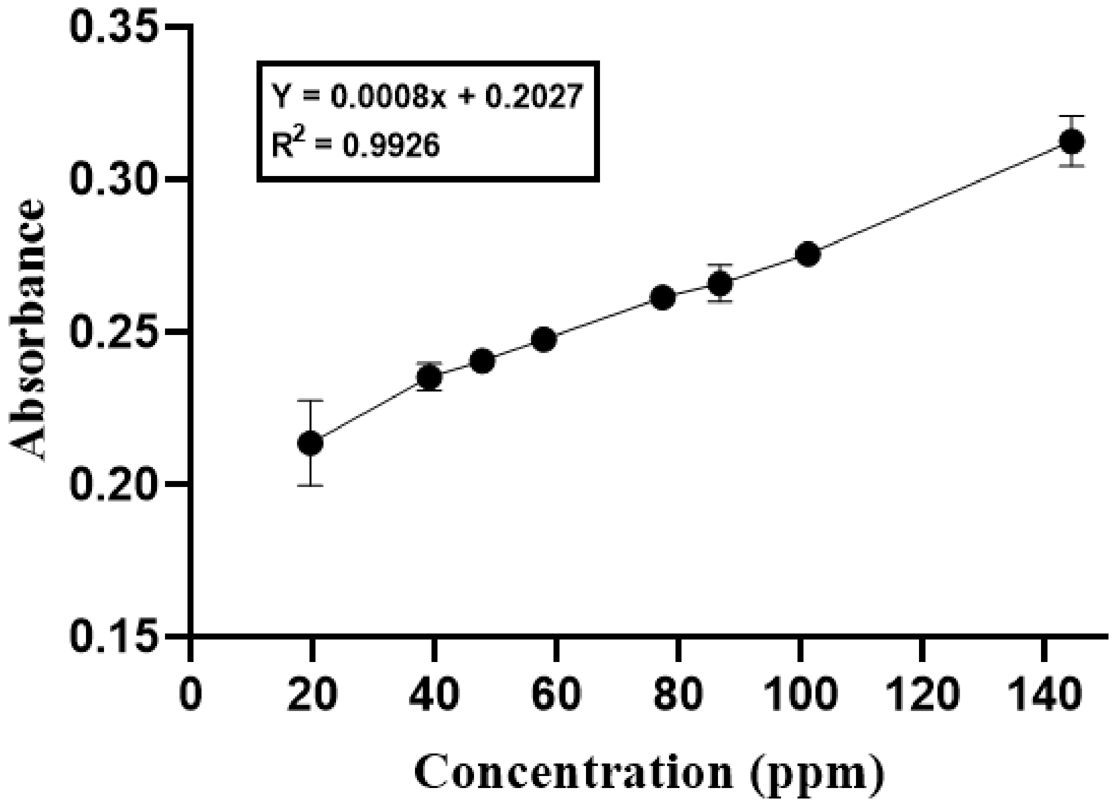

Figure S4: Calibration curve of colistin for calculating the encapsulation efficiency.
